# Trends and outcomes of hospitalized patients with priapism in Germany: results from the GRAND study

**DOI:** 10.1038/s41443-024-00915-5

**Published:** 2024-05-22

**Authors:** Nikolaos Pyrgidis, Gerald B. Schulz, Michael Chaloupka, Yannic Volz, Paulo L. Pfitzinger, Elena Berg, Philipp Weinhold, Friedrich Jokisch, Christian G. Stief, Armin J. Becker, Julian Marcon

**Affiliations:** Department of Urology, University Hospital, LMU, Munich, Germany

**Keywords:** Health care, Diseases

## Abstract

We aimed to provide evidence on the trends and in-hospital outcomes of patients with low- and high-flow priapism through the largest study in the field. We used the GeRmAn Nationwide inpatient Data (GRAND), provided by the Research Data Center of the Federal Bureau of Statistics (2008-2021), and performed multiple patient-level analyses. We included 6,588 men with low-flow and 729 with high-flow priapism. Among patients with low-flow priapism, 156 (2.4%) suffered from sickle cell disease, and 1,477 (22.4%) patients required shunt surgery. Of them, only 37 (2.5%) received a concomitant penile prosthesis implantation (30 inflatable and 7 semi-rigid prosthesis). In Germany, the total number of patients with low-flow priapism requiring hospital stay has steadily increased, while the number of patients with high-flow priapism requiring hospital stay has decreased in the last years. Among patients with high-flow priapism, 136 (18.7%) required selective artery embolization. In men with low-flow priapism, sickle cell disease was associated with high rates of exchange transfusion (OR: 21, 95% CI: 14–31, *p* < 0.001). The length of hospital stay (*p* = 0.06) and the intensive care unit admissions (*p* = 0.9) did not differ between patients with low-flow priapism due to sickle cell disease versus other causes of low-flow priapism. Accordingly, in men with high-flow priapism, embolization was not associated with worse outcomes in terms of length of hospital stay (*p* > 0.9), transfusion (*p* = 0.8), and intensive care unit admission (*p* = 0.5). Low-flow priapism is an absolute emergency that requires shunt surgery in more than one-fifth of all patients requiring hospital stay. On the contrary, high-flow priapism is still managed, in most cases, conservatively.

## Introduction

Priapism is defined as a prolonged erection in the absence of sexual stimulation, which fails to subside [1]. It is generally divided into ischemic (low-flow) and non-ischemic (high-flow) priapism and affects approximately 0.5 per 100,000 males every year [2]. About 95% of all priapism cases are of the ischemic type, which is considered a urological emergency, and is characterized by obstruction of the penile venous outflow, leading to hypoxia, acidosis, and, in turn, to necrosis within the corpora cavernosa [3]. A plethora of causes of ischemic priapism has been identified, including erectile agents, hematological disorders, such as sickle cell disease (SCD), as well as other less common causes such as neurogenic and metabolic disorders, neoplasms, infections, recreational drugs, and other medications [4]. On the contrary, non-ischemic priapism is characterized by increased blood flow due to a fistula between the cavernosal artery and the lacunar spaces of the sinusoidal space, typically occurring after perineal or penile trauma [5].

Ischemic and non-ischemic priapism are different not only in their pathophysiological mechanisms but also in their therapeutic approach [6]. Failure of conservative management of ischemic priapism may require penile shunt surgery [7]. In cases of penile necrosis, an immediate penile prosthesis implantation is recommended [8]. On the contrary, high-flow priapism is rarely an emergency and failure of conservative management of high-flow priapism may require selective arterial embolization. Irrespective of the cause of priapism, hospital admission is advocated for these cases in Germany [9]. Nevertheless, studies exploring the current trends and outcomes of patients admitted into hospital for priapism are lacking [10]. Therefore, we aimed to provide evidence on the trends and in-hospital outcomes of patients with low- and high-flow priapism through the largest study in the field, including only patients from Germany.

## Materials and methods

### GeRmAn Nationwide inpatient Data (GRAND)

The GRAND is a nationwide German dataset (further details under https://www.forschungsdatenzentrum.de/en/health/drg) that contains data on coexisting conditions, procedures, and perioperative outcomes from all hospitalized patients in Germany between 2005 and 2021. These data are sent from all hospitals in Germany to the German Institute for the Hospital Remuneration System to receive their remuneration and include only inpatients. These in-hospital data are subsequently transferred to the Research Data Center of the Federal Bureau of Statistics (Wiesbaden, Germany), where they are stored anonymized. They are coded based on the International Statistical Classification of Diseases and Related Health Problems, 10th revision, German modification (ICD-10-GM), and the German Procedure Classification (OPS). To ensure consistent documentation throughout Germany, coding guidelines are regularly published by the German Institute for Medical Documentation and Information.

For the present analysis, access to the summary results of the GRAND was gained after agreement with the Research Data Center of the Federal Bureau of Statistics (LMU - 4710-2022). Given that the different types of priapism have been coded separately in Germany since 2008, we included all patients requiring hospital stay due to low-flow (ICD-10-GM: N48.30, N48.38, and N48.39) or high-flow (ICD-10-GM: N48.31) priapism from 2008 to 2021. Following the German legislation, approval by an ethics committee and patient informed consent were not necessary. Considering that our research team did not have direct access to patient-level data but only to summary results, the analyses were performed, on our behalf, by the Research Data Center based on R codes developed by our research team. Subsequently, the summary results of the GRAND were sent to our research team for further evaluation (source: Research Data Center of the Federal Bureau of Statistics, DRG Statistics 2005–2021, own calculations). The Research Data Center excluded values with fewer than three baseline characteristics or inpatient complications to ensure anonymity.

### Outcomes and statistical analysis

The primary outcome of the present GRAND analysis was to assess the current trends of patients with low- and high-flow priapism requiring hospital stay in terms of treatment approach. Secondary outcomes included the assessment of in-hospital complications (intensive care unit admission, transfusion) and length of hospital stay in patients with low- and high-flow priapism.

All continuous variables were calculated as median with interquartile range (IQR) and all categorical variables as frequencies with proportions. The corresponding comparisons were performed with the Mann–Whitney test and the chi-squared test. We undertook multivariable logistic and linear regression analyses to assess the effect of SCD-related low-flow priapism and that of selective artery embolization after high-flow priapism on in-hospital outcomes (transfusion, intensive care unit admission, and length of hospital stay). Based on clinical relevance, all models were adjusted for age, diabetes, and obesity. The odds ratios (ORs) with the 95% confidence intervals (CIs) were provided for all logistic models and two-sided p-values lower than 0.05 were considered statistically significant.

## Results

### Baseline characteristics of patients with low-flow priapism

We included a total of 6588 men with low-flow priapism requiring hospital stay from 2008 to 2021. Their median age was 46 years (IQR: 34–57) and 567 (8.6%) patients had diabetes, 415 (6.3%) chronic kidney disease, and 1682 (25.5%) hypertension. Overall, 156 (2.4%) patients developed low-flow priapism due to SCD. Patients with SCD were younger [22 (IQR: 20–26) versus 46 (34–58) years, *p* < 0.001] and presented lower rates of diabetes [567 (8.6%) versus 0, *p* < 0.001], chronic kidney disease [412 (6.4%) versus 3 (1.9%), *p* = 0.04], and hypertension [1,671 (25.6%) versus 11 (7.1%), *p* < 0.001] compared to patients with other causes of low-flow priapism. Accordingly, 1,477 (22.4%) patients with low-flow priapism required shunt surgery. Of them, only 37 (2.5%) received a primary penile prosthesis implantation during the acute admission (30 inflatable and 7 semi-rigid prosthesis). The hospitalization of these patients was more expensive, costing a median of 3616 Euros (IQR: 3217–3986) per patient. Interestingly, 124 (1.9%) patients with low-flow priapism died during hospital stay. Of them, no deaths occurred among patients with SCD, and 16 (1.1%) patients died after shunt surgery. The baseline characteristics of all patients with low-flow priapism are presented in Table 1.

**Table 1:** Baseline characteristics of patients with priapism.

Characteristic	Low-flow priapism, *n* = 6588	Low-flow priapism due to SCD, *n* = 156	Low-flow priapism requiring shunt surgery, *n* = 1477	High-flow priapism, *n* = 729	High-flow priapism requiring selective artery embolization, *n* = 136
Age (years)	46 (34–57)	22 (20–26)	46 (36–56)	39 (26–52)	34 (22–49)
Diabetes	567 (8.6%)	0 (0%)	128 (8.7%)	34 (4.7%)	Less than 3 patients
Chronic kidney disease	415 (6.3%)	3 (1.9%)	95 (6.4%)	26 (3.6%)	Less than 3 patients
Hypertension	1682 (25.5%)	11 (7.1%)	378 (25.6%)	117 (16%)	16 (11.8%)
Cost (Euros)	1864 (852–3614)	3190 (1822–3736)	3616 (3217–3986)	2,233 (1280–3507)	3023 (2793–3731)
Mortality	124 (1.9%)	0 (0%)	16 (1.1%)	5 (0.7%)	0 (0%)
Length of hospital stay (days)	3 (1–7)	4 (1-9)	5 (3–9)	3 (1–7)	4 (2–7)
Transfusion	398 (6%)	57 (37%)	80 (5.4%)	33 (4.5%)	7 (5.1%)
ICU admission	342 (5.2%)	7 (4.5%)	76 (5.1%)	31 (4.3%)	4 (2.9%)

*ICU* intensive care unit, *SCD* sickle cell disease.

### Baseline characteristics of patients with high-flow priapism

We included a total of 729 men with high-flow priapism requiring hospital stay from 2008 to 2021. Their median age was 39 years (IQR: 26–52) and 34 (4.7%) patients had diabetes, 26 (3.6%) chronic kidney disease, and 117 (15.5%) hypertension. Overall, 136 (18.7%) required selective artery embolization due to priapism. Patients undergoing embolization were younger [34 (IQR: 22–49) versus 41 (27–53) years, *p* < 0.001] compared to patients treated conservatively for high-flow priapism. The hospitalization of these patients was more expensive, costing 3023 Euros (IQR: 2793–3731). Interestingly, 5 (0.7%) patients with high-flow priapism died during hospital stay. Of them, no deaths occurred among patients undergoing selective artery embolization. The baseline characteristics of all patients with high-flow priapism are presented in Table 1.

### Annual trends of patients with priapism

**Fig. 1:**
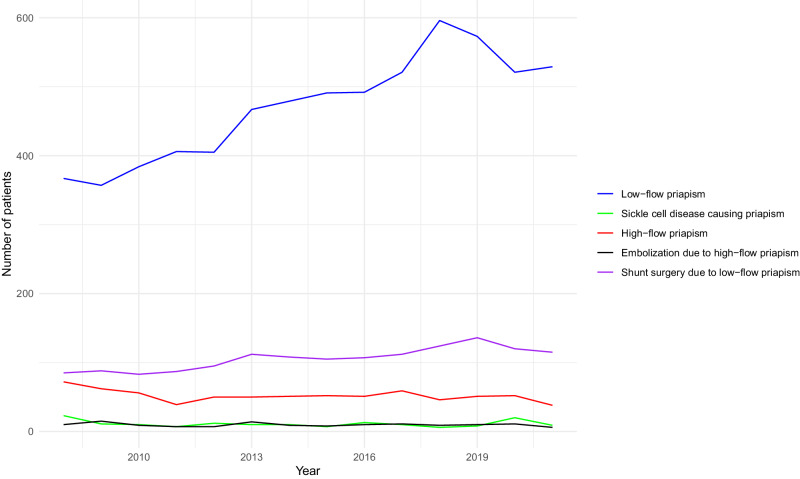
The annual number of patients with priapism.

In Germany, the total number of patients with low-flow priapism requiring hospital stay has steadily increased in the last years from 367 cases in 2008 to 529 cases in 2021. The latter is attributed to other non-SCD causes, given that the total cases of SCD-related low-flow priapism decreased from 23 in 2008 to 9 cases in 2021. Furthermore, the number of patients requiring shunt surgery due to low-flow priapism increased from 85 in 2008 to 115 in 2021. On the contrary, the total number of patients with high-flow priapism requiring hospital stay has decreased in the last years from 72 cases in 2008 to 38 cases in 2021. Still, the number of patients requiring embolization has remained relatively stable from 10 cases in 2008 to 6 cases in 2021. The length of hospital stay did not change during the study period for patients with low- or high-flow priapism. The annual trends of priapism are depicted in Fig. 1.

### In-hospital outcomes of priapism

The median hospital stay due to low-flow priapism was 3 days (IQR: 1–7), and 342 (5.2%) patients required intensive care unit admission. In the multivariate analysis after adjusting for age, diabetes, and obesity, SCD was not associated with longer hospital stay (difference of 2.1 days, 95% CI: −0.1 to 4, *p* = 0.06) or higher rates of intensive care unit admission [7 (4.5%) versus 335 (5.2%), OR: 1, 95% CI: 0.4–1.9, *p* = 0.9] compared to other causes of low-flow priapism. Nevertheless, 57 (37%) patients with low-flow priapism due to SCD were managed with exchanged blood transfusion. As expected, SCD was associated with higher rates of transfusion [57 (37%) versus 341 (5.3%), OR: 21, 95% CI: 14–31, *p* < 0.001]. In patients with high-flow priapism, the median hospital stay was 3 days (IQR: 1–7) and 31 (4.3%) patients required transfusion. In the multivariate analysis after adjusting for age, diabetes, and obesity, selective artery embolization was not associated with longer hospital stay (difference of −0.1 days, 95% CI: −2.9 to 2.6, *p* > 0.9) or higher rates of transfusion [7 (5.1%) versus 26 (4.4%), OR: 1.2, 95% CI: 0.5–2.6, *p* = 0.8] and intensive care unit admission [4 (2.9%) versus 27 (4.6%), OR: 0.7, 95% CI: 0.2–1.8, *p* = 0.5] compared to patients with high-flow priapism treated conservatively. The multivariate regression analysis is displayed in Table 2.

**Table 2:** Multivariable linear and logistic regression analysis for length of hospital stay, transfusion, and ICU admission in patients with low-flow priapism due to SCD, as well as in patients undergoing embolization due to high-flow priapism.

	Length of hospital stay		Transfusion		ICU admissions	
Priapism	Beta	*p*-value	OR	*p*-value	OR	*p*-value
Low-flow						
SCD	2.1 (−0.1, 4.4)	0.06	21 (14, 31)	**<0.001**	1 (0.4, 1.9)	0.9
High-flow						
Selective artery embolization	−0.1 (−2.9, 2.6)	>0.9	1.2 (0.5, 2.6)	0.8	0.7 (0.2, 1.8)	0.5

*ICU* intensive care unit, *OR* odds ratio, *SCD* sickle cell disease.

All models are adjusted for age, diabetes, and obesity. The bold cells indicate statistically significant *p*-values.

## Discussion

The findings of the present study indicate that the incidence of cases with low-flow priapism requiring hospital stay steadily increases in Germany. Accordingly, the incidence of patients with high-flow priapism requiring hospital stay seems to have decreased in the last few years. Still, in Germany, about 10% of all patients requiring hospital stay due to priapism present with high-flow priapism. Interestingly, in contrast to the available literature, in Germany, only a small amount of patients develops SCD-related low-flow priapism. Moreover, more than 20% of all patients with low-flow priapism requiring hospital stay undergo shunt surgery. Nevertheless, only 2.5% of these patients received primary penile prosthesis implantation. Based on our analyses for the in-hospital outcomes, SCD-related low-flow priapism is commonly managed with exchange transfusion, whereas patients undergoing selective artery embolization for high-flow priapism presented similar in-hospital outcomes compared to those managed conservatively.

Even though studies exploring the in-hospital trends of patients with priapism have not yet been reported in the literature, studies on the emergency department trends of priapism from the USA suggest that the incidence of low-flow priapism has increased in the last years [2]. In line with our findings, it seems that the incidence of SCD-related low-flow priapism and high-flow priapism have slightly decreased [11]. The latter is predominantly attributed to the improvements in the management of patients with SCD and those with pelvic injuries [12–14]. Overall, about 10% of all patients presenting to the emergency department with priapism require hospital stay and this proportion is even higher in patients with comorbidities or SCD [15, 16]. In particular, more than 20% of all patients with low-flow priapism in the USA, who were admitted to the hospital, were diagnosed with SCD [17]. Still, based on our findings, this proportion seems to be only 2.4% among patients requiring hospital stay in Germany [18]. The latter might be attributed to the low prevalence of SCD in central European countries. Based on the previous notion, a nationwide study from Denmark identified only three cases of SCD-related priapism from 1980 to 2016 [19].

It should be highlighted that the present study demonstrates that the nationwide trends from Germany diverge from the current guideline recommendations [1, 10]. More specifically, even though exchange transfusions should be implemented only with caution in patients with SCD-related low-flow priapism due to their serious adverse events in some cases [20], more than one-third of all patients with SCD-related low-flow priapism requiring hospital stay was managed with exchange transfusions. Accordingly, although primary penile prosthesis implantation is advocated for men with delayed or refractory low-flow priapism requiring shunt surgery [21], only 37 prostheses have been placed in Germany for this indication. Nevertheless, it should be noted that priapism is rarely the cause of penile prosthesis implantation in Germany [22].

Studies exploring the current trends and outcomes of high-flow priapism are also lacking. It seems that high-flow priapism should be initially managed conservatively [23]. After failure of conservative treatment, selective artery embolization is recommended [24]. In line with our findings, available literature suggests that selective artery embolization is a highly safe and effective procedure with rare short- and long-term complications [25, 26]. Moreover, previous studies indicate that patients with persisting high-flow priapism undergoing selective artery embolization present better functional outcomes compared to those treated conservatively [27]. Based on the previous notion, it should be noted that erectile function can be maintained to the pre-interventional levels in an important amount of patients and re-embolization rates are considered relatively low [28]. Still, it seems that selective artery embolization for high-flow priapism is not yet widely implemented in Germany.

Even though we present, to the best of our knowledge, the largest analysis on patients with priapism requiring hospital stay, our findings should be interpreted with caution due to some important limitations. No data on those discharged after treatment in the emergency department are available. Still, it should be noted that an important amount of patients requiring therapy for priapism receive in hospital treatment due to the German reimbursement system based on the DRG. Furthermore, all analyses were based on retrospective, administrative data, and, thus, are prone to coding errors, and selection bias. Importantly, the patients’ laboratory findings, the duration of symptoms, previous priapism episodes, as well as the selected conservative approach for low- and high-flow priapism are not presented in the GRAND study. Moreover, no information on the preferred surgical technique for shunting (anterior or posterior), as well as the as well as on the type of the selected shunting technique (T-shunt, tunneling, other techniques) are provided. Similarly, data after hospital discharge, readmission and regression rates, re-embolization rates, functional outcomes such as erectile dysfunction, and long-term follow-up visits are not collected. Given that the Research Data Center excluded values with fewer than three events to ensure anonymity, we could not assess further in-hospital outcomes or perform additional subgroup analyses. Importantly, all data derive exclusively from Germany and, thus, may not be extrapolated to other healthcare systems. Still, aiming to overcome these limitations, our holistic approach, combined with the high-volume analyses, leads to solid conclusions for the trends in the management of priapism.

## Conclusion

Low-flow priapism is an absolute emergency that requires shunt surgery in more than one-fifth of all patients admitted to hospital. In Germany, despite guideline recommendations, primary placement of a penile prosthesis after shunt surgery is not commonly preferred, whereas exchange transfusions are often implemented in patients with SCD. Still, SCD does not seem to be a leading cause of low-flow priapism in Germany. On the contrary, high-flow priapism is managed, in most cases, conservatively. Nevertheless, patients requiring selective artery embolization present favorable in-hospital outcomes.

## Data Availability

The data supporting this study’s findings are available from the corresponding author upon reasonable request.
